# Predictors and barriers to breastfeeding in north of Jordan: could we do better?

**DOI:** 10.1186/s13006-017-0140-y

**Published:** 2017-12-08

**Authors:** Wasim Khasawneh, Ayat Abdelrahman Khasawneh

**Affiliations:** 1 Department of Pediatrics, Jordan University of Science and Technology, PO Box 3030, Irbid, 22110 Jordan; 2 Department of Community Medicine, Jordan Royal Medical Services, Prince Rashid Military Hospital, Aidoun, Irbid, 22110 Jordan

## Abstract

**Background:**

Despite the ongoing recommendations for breastfeeding, we continue to see a decrease in exclusive breastfeeding among Jordanian women during infant follow up visits at the pediatric outpatient clinic. The purpose of our study is to determine the prevalence, predictors and barriers to exclusive breastfeeding in north of Jordan.

**Methods:**

We conducted a cross-sectional survey involving mothers with infants six to twelve months old, at two hospitals in Irbid city in north of Jordan, between December 2016 and March 2017. Questions included demographics, feeding pattern, and reasons for non-exclusive breastfeeding.

**Results:**

Five hundred women were included. Twenty four percent of women were employed and 87% initiated breastfeeding within three hours of birth. The proportion of women with any breastfeeding and exclusive breastfeeding at six months was 76 and 33%. After multivariate logistic regression analysis, predictors of exclusive breastfeeding at six months include the mother’s previous experience (Adjusted Odds Ratio [AOR] 7.9, 95% CI 4.69, 13.36) and multiparity (AOR 2.26, 95% CI 1.2, 4.28), while barriers include maternal employment (AOR 0.4, 95% CI 0.22,0.72), Cesarean delivery (AOR 0.55, 95% CI 0.35, 0.86) and infant’s hospitalization (AOR 0.44, 95% CI 0.23,0.82). Inadequate breastmilk supply and short maternity leave were the main reported reasons for non-exclusive breastfeeding.

**Conclusions:**

In north of Jordan, the majority of women initiate breastfeeding, half practice exclusive breastfeeding after birth while one third continue for six months, particularly those with previous experience. Cesarean delivery and infant’s hospitalization, together with maternal employment are among the main barriers. Implementing educational programs and lactation consultant counselling together with work environment support, should be helpful to improve the breastfeeding practice among Jordanian women.

## Background

Breastfeeding is superior to other modes of enteral nutrition for infants in the first year of life and has multiple benefits for the infant and the nursing mothers [1–4]. This fact has been repeatedly supported by the World Health Organization (WHO), the American Academy of Pediatrics (AAP), and multiple international agencies [5, 6].

Before the twenty-first century, it was traditional in Jordan and other developing countries in the region for mothers to exclusively breastfeed their children for at least 12 months [7]. Recently, the rate of female education and employment during childbearing age increased [8, 9] and this factor, although not well studied and supported in Jordan, might have negatively affected the practice of breastfeeding partly due to mother’s short maternity leave, and partly due to limited support in the work environment to practice breastfeeding and pumping. The pattern of breastfeeding in Jordan was studied in the past [10–12]. A paper published in 2006 reviewing the practice of breastfeeding in 2003 reported that 58% of the 344 studied women practice exclusive breastfeeding (EBF), of whom one third continued for 6–12 months of life [11]. In their study, the authors found that maternal employment and cesarean delivery were the main barriers against exclusive breastfeeding. Employed women were more likely not to EBF compared with unemployed women (OR 3.34, 95% CI 1.6, 6.98) and women who had cesarean delivery were more likely not to practice EBF (OR 2.36, 95% CI 1.17,4.78) [11].

On the other hand, the rate of breastfeeding and EBF has been rising in the developed countries. In USA, the Centers for Disease Control and prevention (CDC) annually publish a report card about breastfeeding practice in all states. In the 2014 report, the proportion of ever breastfeeding mothers was about 80% in most of the states with 15–28% of EBF at six months [13, 14]. The educational programs about breastfeeding that start during pregnancy and continue after delivery, together with the help of lactation consultants and special supportive facilities in the workplaces, have contributed a lot to this achievement. Those practices in the US were supported by public health campaigns such as the U.S. Healthy People 2010 and 2013 initiatives [15].

We therefore conducted this study to shed light on the practice of breastfeeding in north of Jordan to determine the prevalence of EBF, identify the predictors and barriers, and make to suggestions towards educating, counselling and supporting mothers in order to improve the rate of EBF to match the WHO recommendations.

## Methods

This face-to-face cross-sectional structured questionnaire survey was conducted at the outpatient pediatric clinic at King Abdullah University Hospital (KAUH), a tertiary academic hospital of Jordan University of Science and Technology, and at Prince Rashid Military Hospital (PRMH) in the city of Irbid in the north of Jordan, during the period December 2016 to March 2017.

These two hospitals in the north of Jordan provide health services to more than two million of the Jordanian population with an annual number of deliveries approaching thirteen thousands per year in both places combined, which constitute about 30% of the total annual births in the region [16]. The majority of Irbid city inhabitants are middle class families with health insurance coverage through employment. Due to the large number of births and limited resources, both hospitals lack baby-friendly postpartum units.

### Participants and data collection

Mothers with infants six months to one year of age were randomly selected at their visit to the outpatient department during the study period. Using the Open-Epi program, and based on a population size of 13,000 assuming an estimated six month EBF prevalence of 50% with a 95% confidence interval, we calculated a required sample size of 468 with 5% margin of error and an expected response rate of 80%. To ensure our randomly chosen sample represented the regional population, a descriptive analysis of the sociodemographic data of the studied participants, including maternal age, parity, gestational age, employment status and previous experience, were compared among both hospitals. Using median ± SD for continuous variables and proportions for nominal variables and were found to be quite similar.

After both authors received training and agreed on the interview instructions and technique, either one discussed the survey questionnaire with the mothers. Verbal consent was obtained from the study participants.

The survey questionnaire included demographic characteristics about the mothers and their infants, maternal employment, duration of maternity leave, previous experience with breastfeeding, and patterns of feeding at birth, one month, six months and after six months of age.

Mothers who did not practice EBF were asked a direct question about the reason and given the chance to choose one or more from the list of potential reasons.

Exclusive breastfeeding was defined as the infant was fed only breast milk without any other enteral intake except for medications and vitamins. In our study, we referred to breastfeeding initiation if fed within the first three hours after delivery, while EBF at birth refers to the first 48 h after delivery.

### Data analysis

Statistical analysis was performed using IBM SPSS statistics version 23 and Microsoft Office Excel 2013. Descriptive statistics was used to summarize the socio-demographic characteristics of the study participants and the prevalence of exclusive breastfeeding. After identifying the factors affecting EBF using univariate regression analysis, a multivariate logistic regression analysis was performed to identify factors associated with EBF within 48 h after birth and at six months. With *p* ≤ 0.05 chosen as the level of statistical significance, Odds ratios (OR) and their 95% confidence intervals (CI) were calculated and reported.

## Results

A total of 620 eligible women were approached to participate in this survey during the study period. Five hundred were included in the analysis as 20 declined to participate and 100 were excluded since their babies were younger than six months at the time of interview. Table 1 summarizes the demographics and 62% of women were delivered vaginally and 38% by cesarean section. The rate of maternal employment was 24% with an average maternity leave of 70 to 90 days. Eighty nine percent of the included babies were born at 37 week gestation or more. Twenty three percent of the women were primiparous, and 41% of mothers had an experience of EBF with their previous children.

**Table 1:** Maternal and infant characteristicsª

Characteristic	*n* = 500 (%)
Age (years)	
≤ 30	311 (62)
> 30	189 (38)
Parity	
1	115 (23)
> 1	385 (77)
Previous experience with BF 4–6 months	
Exclusive BF	205 (41)
Partial BF	121 (24)
No BF	59 (12)
N/A	115 (23)
Mode of delivery	
Vaginal	309 (62)
C/S	191 (38)
Employed	
No	380 (76)
Yes	120 (24)
Gestational age	
< 37	55(11)
≥ 37	445(89)
Gender	
Male	281 (56)
Female	219 (44)
Multiple gestation	
No	488 (97.6)
Yes	12 (2.4)
Hospital admission	
Direct	85 (17)
Home	22 (4)
No	393 (88)
Neonatal jaundice	
Yes	176 (35)
No	324 (65)

^a^Data from King Abdullah University Hospital and Prince Rashid Hospital Irbid, Jordan 2017

*BF* Breastfeeding, *C/S* Cesarean section

The proportion of mothers who initiate BF within three hours after birth was 87%. The proportion of EBF at one month and six months of life was 47 and 33% respectively. After the age of six months, nearly half of the mothers were not breastfeeding at all, and less than 10% were practicing EBF (Table 2).

**Table 2:** Pattern of breastfeeding in the first year of life^a^

Pattern of BF	At birth® *n* = 500 (%)	At one month *n* = 500 (%)	At 6 months *n* = 500 (%)	> 6 months *n* = 500 (%)
Exclusive	253 (51)	234 (47)	167 (33)	47 (9)
Partial	181 (36)	192 (38)	213 (43)	202 (41)
None	66 (13)	74 (15)	120 (24)	251 (50)

*BF* Breastfeeding, *At birth®* Refers to 48 h after birth

^a^Data from King Abdullah University Hospital and Prince Rashid Hospital Irbid, Jordan 2017

As shown in Table 3, previous experience of EBF was associated with a higher proportion of EBF in the first 48 h after birth (75% vs 34%), while Cesarean delivery and infant’s admission to the hospital were associated with a lower proportion. The proportion of women who practice EBF within 48 h after birth was not affected by maternal age or parity although higher EBF was noticed among multiparous mothers. Maternal employment status did not affect the proportion of EBF in the first 48 h after birth. However, at six months, the proportion of EBF was much higher among non-employed mothers (39% vs 15%).

**Table 3:** Predictors and barriers to exclusive breastfeeding^a^

Variable	EBF at birth (*n =* 253) n (%)	*p* value	Adjusted OR 95% CI	EBF at 6 mon (*n =* 167) n (%)	*p* value	Adjusted OR 95% CI
Mother’s age (years)						
≤ 30 (313)	151 (48)	0.53	0.87 (0.57,1.34)	102 (33)	0.78	0.93 (0.59,1.50)
> 30 (187)	102 (54)			65 (35)		
Parity						
Multi (385)	209 (54)	0.25	1.36 (0.81,2.28)	140 (36)	0.01	2.26 (1.2,4.28)
Primi (115)	44 (38)			27 (24)		
Previous EBF						
Yes (204)	152 (75)	0.0001	5.98 (3.78,9.43)	117 (57)	0.0001	7.91 (4.69,13.36)
No (296)	101 (34)			50 (17)		
Mother’s employment						
Yes (120)	49 (41)	0.67	0.9 (0.57,1.4)	18 (15)	0.002	0.4 (0.22,0.72)
No (380)	204 (54)			149 (39)		
Mode of delivery						
C/S (191)	79 (41)	0.005	0.56 (0.37,0.84)	46 (24)	0.009	0.55 (0.35,0.86)
Vaginal (309)	174 (56)			121 (39)		
NICU admission						
Yes (85)	30 (35)	0.01	0.5 (0.29,0.85)	16 (19)	0.01	0.44 (0.23,0.82)
No (415)	223 (54)			151 (36)		

*EBF* Exclusive Breastfeeding. EBF at birth refers to first 48 h after birth, *C/S* Cesarean section, *NICU* Neonatal Intensive Care Unit

^a^Data from King Abdullah University Hospital and Prince Rashid Hospital Irbid, Jordan 2017

Adjusted OR refers to multivariate logistic regression model

After identifying the factors affecting EBF using univariate regression analysis, a multivariate logistic regression analysis was performed to identify factors associated with EBF within 48 h after birth and at six months. Mother’s previous experience (AOR 7.9, 95% CI 4.69, 13.36) and multiparity (AOR 2.26, 95% CI 1.2, 4.28) were the main predictors of exclusive breastfeeding at six months, while maternal employment (AOR 0.4, 95% CI 0.22,0.72), Cesarean delivery (AOR 0.55, 95% CI 0.35, 0.86) and infant’s hospitalization (AOR 0.44, 95% CI 0.23,0.82) were the main barriers (Table 3).

The main reported reason for non-exclusive breastfeeding was inadequate milk supply (53%), which is a subjective perception made by the mothers and their families. Sixty five percent of the employed mothers stopped breastfeeding completely or partially at the end of their maternity leave. Mothers reported the lack of enough time and absence of worksite private places to pump during the work hours to be the main reasons. In addition, the inability to have their nursing infants accompany them to the work place in the absence of local daycare centers has limited their opportunity to continue breastfeeding (Table 4).

**Table 4:** Reasons for non-exclusive breastfeeding^a^

Causes	*n* = 333 (%)
Inadequate supply	175 (53)
End of maternity leave	77 (23)
Baby’s illness/ hospitalization	45 (14)
Maternal illness	35 (11)
Latching difficulties	17 (5)
Other maternal causes:	
Breast issues	18 (5.4)
Getting pregnant	11 (3.3)
Use of contraceptives	2 (0.6)
Other infant causes:	
Multiple gestation	11 (3.3)
Jaundice	8 (2.4)
Poor weight gain	5 (1.5)

^a^Data from King Abdullah University Hospital and Prince Rashid Hospital Irbid, Jordan 2017

## Discussion

Despite the bulk of evidence emphasizing the benefits of EBF in the first six months of life, women continue to practice differently. In our study, the prevalence of EBF is positively associated with previous EBF experience but negatively associated with cesarean delivery and maternal infant admission to the hospital. Exclusive breastfeeding at six months was also affected by parity and maternal employment. Eighty seven percent of mothers initiate breastfeeding after birth but nearly 40% also supplement their infants with formula.

The continuation of breastfeeding (76%) and EBF (33%) declined by the age of six months, particularly among employed women. These numbers are consistent with studies from other countries in the Middle East region, but remain far from the target set by the WHO, AAP and other international health agencies [5, 6].

Breastfeeding practice in Jordan was studied in the past. In 2006, Khasawneh et al. reported that 89% of women initiate breastfeeding after birth, the proportion of women who practice EBF was 58%, of whom one third did so for six months [11]. About two thirds of the mothers participated in that study continued to breastfeed for more than one year [11]. The national data about breastfeeding and EBF are not well reported from the Arab World and most data are collected by cross-sectional studies. In Saudi Arabia, the rate of initiation of breastfeeding among Saudi mothers was around 92% [17], compared with 57% in Qatar [18], and 98% in United Arab Emirates [19]. The national status and progress of infant nutrition including breastfeeding patterns in Saudi Arabia was reported as a part of the World Breastfeeding Trend Initiative in 2014 [20]. Authors reported the rate of EBF of 44% and focused on the indicators for a better breastfeeding practice including Baby Friendly units and improvement in maternal employment policies [20].

Our study reviewed the practice of breastfeeding and pointed out some demographic and social characteristics that predict success or act as barriers to EBF. Besides multiparity, the main predictive factor for success of EBF was the mother’s own past experience with EBF in her previous children, and nearly two thirds of mothers who EBF their older children do so with their current child. This finding is not unexpected as most of the difficulties in breastfeeding are usually reported in young and nulliparous mothers and these mothers need to be a target group, with better education and support about the benefits and techniques of breastfeeding to improve their future experiences [21, 22]. We also found that a Cesarean delivery and an infant’s admission to the hospital were among the major barriers to initiation and continuation of EBF.

Our findings support other studies in the region; Al Junaid concluded that multiparity was among the major factors associated with high prevalence of breastfeeding in Saudi Arabia [23]. More recently, another cross-sectional study involving 671 mothers from Saudi Arabia published by Alzaheb about the factors affecting early initiation of breastfeeding, found that delivering a preterm or low birth weight infant as well as cesarean section deliveries were strong barriers to breastfeeding, and the author recommended establishing hospital policies and health staff training as a vital measure in improving breastfeeding practice [24].

Nearly half of our babies receive their first feeding in the newborn nursery using a standard infant formula. The practice of skin-to-skin care immediately after birth is not optimal at our institutions and is negatively impacted by the limited space in labor and delivery units, and the unavailability of epidural anesthesia in the majority of cases. Those factors together with the absence of Baby-Friendly units reduce the chance to initiate breastfeeding in the first hour of life. Studies have shown that skin-to-skin care in the immediate postnatal period enhances the EBF practice and increases mother’s milk supply [25, 26]. The same recommendation was highlighted in the NICHD statement about the benefits of breastfeeding [27].

A large number of studies from Ethiopia and other resource limited countries in Africa show that colostrum feeding as the first enteral feeding received by an infant promotes EBF and improves infants’ survival by improving oral suckling skills and facilitating milk production [28, 29].

Findings from previously published reports in Western countries which examined the predictors for success of breastfeeding, showed that maternal age and education as well as healthcare providers’ support were strongly associated with a higher rate of breastfeeding initiation [21, 30, 31]. Bailey reported a better success rate in primiparous mothers [15]. Many studies focused on the link between maternal smoking and breastfeeding [15]; the prevalence of smoking among women in our study population was very negligible and so not included in our analysis.

The other barrier to EBF in our study is the employment of the mother. This challenge has been extensively studied in literature [32–35] with strong evidence that maternal employment post birth is associated with shorter breastfeeding duration, although not with decreased initiation of breastfeeding. In our participants, the percentage of women who initiate breastfeeding at birth was comparable between employed and non-employed mothers. However, the rate of EBF was different at six months of age.

Nowadays, we see a higher rate of employment among women in Jordan and other developing countries. Improving the socioeconomic status of young women can have contradictive effects on family rising and infant feeding practices. With a better education, mothers can learn more about the benefits of EBF and its impact on their child’s health and long-term outcome. However, employed mothers have less time to spend with their children at the end of maternity leave, and with a better financial status can easily afford to buy infant formula, which negatively impacts EBF rates. In addition, the short maternity leave and the lack of supportive measures in the work environment discourage mothers from practicing EBF and lower their efforts to continue breastfeeding [36–38].

The U.S. studies about the employment status as a predictive factor of breastfeeding have shown that EBF is higher among non-employed and part time workers. The breastfeeding rates are higher for working mothers when there is access to a flexible workplace environment, when lactating women are offered more support from employers and co-workers, and given enough time and place to nurse their children or express their milk [36].

One of the main reasons for not EBF in our study is the mother’s perception and belief of an inadequate milk supply based on infant’s crying and being unsettled after feeding. Mothers in this study receive their support and encouragement solely from their families. The lack of educational programs about breastfeeding during pregnancy and the absence of lactation consultant services and postnatal support programs could have contributed to this finding [39, 40]. Other barriers to breastfeeding and EBF described in our study including maternal illness, latching difficulties and infant’s hospitalization were consistent with what has been reported by others [29, 34].

Potential solutions to overcome the challenges to exclusive breastfeeding include the establishment of counselling and educational programs during pregnancy to prepare the mothers mentally and emotionally to breastfeed their infants.

Working with the hospital administration to create Baby-Friendly units and apply the Ten Steps to Successful Breastfeeding as endorsed by the World Baby Friendly Initiative is considered the cornerstone in improving the rate of EBF. These steps include written policies, staff training, maternal counselling, and avoiding mother-infant separation in the immediate postnatal period [1]. The hiring lactation consultants is needed to spend enough time with postpartum mothers to teach the right technique for breastfeeding, and to help especially the young and primiparous mothers, to overcome the difficulties they might face in the first few days. These consultants should also be available and pivotal during follow up visits at the outpatient clinic. We should also focus on endorsing the International Code for controlling the Marketing and distribution of Breastmilk Substitutes [41].

Without greater support for employed mothers who want to breastfeed, the continued negative impact on continuation of breastfeeding will likely increase with the increasing rate of employment among young women. Many of the challenges that face this group of mothers can be minimized with a judicious utilization of space and policy flexibility from employers, together with support from coworkers, family, and friends. Implementing strategies and policies to create daycare centers and lactation programs in the workplace environment should improve breastfeeding practices and positively inspire employed mothers to better achieve at work and at childcare.

Our study is not without limitations. The major limitation is its cross-sectional design that makes conclusions about predictors and barriers to EBF not very accurate. To minimize the recall bias usually associated with cross-sectional study designs, we limited the age of the involved children to less than one year. Another limitation is the inability to infer conclusions applicable to the whole Jordanian population since our sample does not represent the whole country. In addition, we deviated from the WHO definition of early initiation of breastfeeding within the first hour after birth and used three hours instead in our study population, and this might have falsely elevated the proportion of breastfeeding initiation but it has no effect on the exclusive breastfeeding outcome at six months of life.

## Conclusions

In summary, the rate of exclusive breastfeeding among Jordanian women is below the WHO recommendation. Challenges in initiating and maintaining EBF are consistent with the rest of the world. Encouraging breastfeeding is a primary health promotion strategy, collaboration between health professionals, social services, workplaces, and the community is required to improve the breastfeeding experience for women and their babies.

## Abbreviations

AAP: American Academy of Pediatrics
EBF: Exclusive breastfeeding
IRB: Institutional Board Review
NICHD: National Institute of Child Health and Human Development
WHO: World Health Organization

## References

[1] Victora CG, Bahl R, Barros AJD, França GVA, Horton S, Krasevec J (2016). Breastfeeding in the 21st century: epidemiology, mechanisms, and lifelong effect. Lancet.

[2] Sankar MJ, Sinha B, Chowdhury R, Bhandari N, Taneja S, Martines J (2015). Optimal breastfeeding practices and infant and child mortality: a systematic review and meta-analysis. Acta Paediatr.

[3] Kowalewska-Kantecka B (2016). Breastfeeding - an important element of health promotion. Dev Period Med.

[4] The Lancet. Breastfeeding: achieving the new normal. Lancet. 2016;387:404. Available from: http://linkinghub.elsevier.com/retrieve/pii/S0140673616002105 10.1016/S0140-6736(16)00210-5 26869549

[5] WHO. Early initiation of breastfeeding to promote exclusive breastfeeding. World Health Organization; 2017 [cited 2017 Mar 28]; Available from: http://www.who.int/elena/titles/early_breastfeeding/en/

[6] Eidelman A, Schanler R. Breastfeeding and the use of human milk. Pediatrics. 2012;129:e827–41. Available from: http://pediatrics.aappublications.org/cgi/doi/10.1542/peds.2011-3552 10.1542/peds.2011-3552 22371471

[7] Akin JS, Bilsborrow RE, Guilkev DK, Popkin BM. Breast-feeding patterns and determinants in Jordan 1984 [cited 2017 Jul 26]; Available from: http://pdf.usaid.gov/pdf_docs/PNAAY732.pdf 12280550

[8] Trends in demographic and health indicators in Jordan data from the 1990–2012 Jordan population and family health surveys. [cited 2017 Sep 18]; Available from: https://dhsprogram.com/pubs/pdf/TR8/TR8.pdf

[9] Majcher-Teleon A. Women and work in Jordan case study of tourism and ICT sectors. 2009 [cited 2017 Sep 20]; Available from: http://www.silviacambie.com/wp-content/uploads/2010/01/womenwork-in-jordan.pdf

[10] Al-Akour N, Khassawneh M, Khader YS, Ababneh A, Haddad AM (2010). Factors affecting intention to breastfeed among Syrian and Jordanian mothers: a comparative cross-sectional study. Int Breastfeed J.

[11] Khassawneh M, Khader Y, Amarin Z, Alkafajei A (2006). Knowledge, attitude and practice of breastfeeding in the north of Jordan: a cross-sectional study. Int Breastfeed J.

[12] Oweis A, Tayem A, Froelicher ES (2009). Breastfeeding practices among Jordanian women. Int J Nurs Pract.

[13] N Center for Chronic Disease Prevention, Promotion H, of Nutrition D, Activity P. Breastfeeding report card, progressing toward national breastfeeding goals: United States/2016. [cited 2017 Sep 17]; Available from: https://www.cdc.gov/breastfeeding/pdf/2016breastfeedingreportcard.pdf

[14] C for Disease Control. Breastfeeding report card: United States, 2014. 2014 [cited 2017 Sep 19]; Available from: https://www.cdc.gov/breastfeeding/pdf/2014breastfeedingreportcard.pdf

[15] Bailey BA, Wright HN (2011). Breastfeeding initiation in a rural sample: predictive factors and the role of smoking. J Hum Lact.

[16] The Hashemite Kingdom of Jordan social trends in Jordan social trends in Jordan. 2006 [cited 2017 Sep 17]; Available from: http://www.dos.gov.jo/dos_home_e/Social Trends in Jordan.pdf.

[17] El Mouzan MI, Al Omar AA, Al Salloum AA, Al Herbish AS, Qurachi MM (2009). Trends in infant nutrition in Saudi Arabia: compliance with WHO recommendations. Ann Saudi Med.

[18] Al-Kohji S, Said HA, Selim NA (2012). Breastfeeding practice and determinants among Arab mothers in Qatar. Saudi Med J.

[19] Radwan H (2013). Patterns and determinants of breastfeeding and complementary feeding practices of Emirati mothers in the United Arab Emirates. BMC Public Health.

[20] The World Breastfeeding Trends Initiative (WBTi). [cited 2017 Sep 17]; Available from: www.worldbreastfeedingtrends.org.

[21] Kitano N, Nomura K, Kido M, Murakami K, Ohkubo T, Ueno M (2016). Combined effects of maternal age and parity on successful initiation of exclusive breastfeeding. Prev Med Rep.

[22] Hackman NM, Schaefer EW, Beiler JS, Rose CM, Paul IM (2015). Breastfeeding outcome comparison by parity. Breastfeed Med.

[23] Al Juaid DAM, Binns CW, Giglia RC (2014). Breastfeeding in Saudi Arabia: a review. Int Breastfeed J.

[24] Alzaheb RA (2016). Factors associated with the initiation of breastfeeding within the first 48 hours of life in Tabuk, Saudi Arabia. Int Breastfeed J.

[25] Moore ER, Bergman N, Anderson GC, Medley N. Early skin-to-skin contact for mothers and their healthy newborn infants. In: Moore ER, editor. Cochrane Database Syst Rev. 2016;11:CD003519. Available from: http://www.ncbi.nlm.nih.gov/pubmed/27885658 10.1002/14651858.CD003519.pub4 PMC6464366 27885658

[26] Dashti M, Scott JA, Edwards CA, Al-Sughayer M (2010). Determinants of breastfeeding initiation among mothers in Kuwait. Int Breastfeed J.

[27] Crenshaw JT (2014). Healthy birth practice #6: keep mother and baby together- It’s best for mother, baby, and breastfeeding. J Perinat Educ.

[28] Liben ML, Gemechu YB, Adugnew M, Asrade A, Adamie B, Gebremedin E (2016). Factors associated with exclusive breastfeeding practices among mothers in Dubti town, afar regional state, northeast Ethiopia: a community based cross-sectional study. Int Breastfeed J.

[29] Seid AM, Yesuf ME, Koye DN (2013). Prevalence of exclusive breastfeeding practices and associated factors among mothers in Bahir Dar city, Northwest Ethiopia: a community based cross-sectional study. Int Breastfeed J.

[30] Sutherland T, Pierce CB, Blomquist JL, Handa VL (2012). Breastfeeding practices among first-time mothers and across multiple pregnancies. Matern Child Health J.

[31] Li L, Zhang M, Scott JA, Binns CW (2004). Factors associated with the initiation and duration of breastfeeding by Chinese mothers in Perth, Western Australia. J Hum Lact.

[32] Baker M, Milligan K (2008). Maternal employment, breastfeeding, and health: evidence from maternity leave mandates. J Health Econ.

[33] Guttman N, Zimmerman DR (2000). Low-income mothers’ views on breastfeeding. Soc Sci Med.

[34] Taveras EM, Li R, Grummer-Strawn L, Richardson M, Marshall R, Rêgo VH (2004). Opinions and practices of clinicians associated with continuation of exclusive breastfeeding. Pediatrics.

[35] Arthur CR, Saenz RB, Replogle WH (2003). The employment-related breastfeeding decisions of physician mothers. J Miss State Med Assoc.

[36] Ryan AS, Zhou W, Arensberg MB (2006). The effect of employment status on breastfeeding in the United States. Women’s Heal Issues.

[37] Dennis C-L (2002). Breastfeeding initiation and duration: a 1990–2000 literature review. J Obstet Gynecol Neonatal Nurs.

[38] Chuang C-H, Chang P-J, Chen Y-C, Hsieh W-S, Hurng B-S, Lin S-J (2010). Maternal return to work and breastfeeding: a population-based cohort study. Int J Nurs Stud.

[39] Wood NK, Sanders EA. Mothers with perceived insufficient milk. West J Nurs Res. 2017;19394591668755. Available from: http://www.ncbi.nlm.nih.gov/pubmed/28322655. 10.1177/0193945916687552 28322655

[40] Vural F, Vural B (2017). The effect of prenatal and postnatal education on exclusive breastfeeding rates. Minerva Pediatr.

[41] WHO | Regulation of marketing breast-milk substitutes. WHO [Internet]. World Health Organization; 2016 [cited 2017 Jul 27]; Available from: http://www.who.int/elena/titles/regulation_breast-milk_substitutes/en/

